# SIV replication is directly downregulated by four antiviral miRNAs

**DOI:** 10.1186/1742-4690-10-95

**Published:** 2013-08-29

**Authors:** Jeanne M Sisk, Kenneth W Witwer, Patrick M Tarwater, Janice E Clements

**Affiliations:** 1 Department of Molecular and Comparative Pathobiology, Edward D. Miller Research Building, The Johns Hopkins University School of Medicine, 733 N Broadway, Baltimore, MD 21205, USA; 2 Division of Biostatistics & Epidemiology, Texas Tech University Health Science Center, El Paso, TX, USA; 3 Department of Neurology, Edward D. Miller Research Building, The Johns Hopkins University School of Medicine, 733 N Broadway, Baltimore, MD 21205, USA; 4 Department of Pathology, Edward D. Miller Research Building, The Johns Hopkins University School of Medicine, 733 N Broadway, Baltimore, MD 21205, USA

**Keywords:** MicroRNA, MiR-29a, MiR-29b, MiR-9, MiR-146a, IFNb, TNFa, HIV, SIV, Macrophages

## Abstract

**Background:**

Host cell microRNAs (miRNAs) have been shown to regulate the expression of both cellular and viral RNAs, in particular impacting both Hepatitis C Virus (HCV) and Human Immunodeficiency Virus (HIV). To investigate the role of miRNAs in regulating replication of the simian immunodeficiency virus (SIV) in macrophage lineage cells, we used primary macrophages to study targeting of SIV RNA by miRNAs. We examined whether specific host miRNAs directly target SIV RNA early in infection and might be induced via type I interferon pathways.

**Results:**

miRNA target prediction programs identified miRNA binding sites within SIV RNA. Predicted binding sites for miRs-29a, -29b, -9 and -146a were identified in the SIV Nef/U3 and R regions, and all four miRNAs decreased virus production and viral RNA expression in primary macrophages. To determine whether levels of these miRNAs were affected by SIV infection, IFNβ or TNFα treatments, miRNA RT-qPCR assays measured miRNA levels after infection or treatment of macrophages. SIV RNA levels as well as virus production was downregulated by direct targeting of the SIV Nef/U3 and R regions by four miRNAs. miRs-29a, -29b, -9 and -146a were induced in primary macrophages after SIV infection. Each of these miRNAs was regulated by innate immune signaling through TNFα and/or the type I IFN, IFNβ.

**Conclusions:**

The effects on miRNAs caused by HIV/SIV infection are illustrated by changes in their cellular expression throughout the course of disease, and in different patient populations. Our data demonstrate that levels of primary transcripts and mature miRs-29a, -29b, -9 and -146a are modulated by SIV infection. We show that the SIV 3′ UTR contains functional miRNA response elements (MREs) for all four miRNAs. Notably, these miRNAs regulate virus production and viral RNA levels in macrophages, the primary cells infected in the CNS that drive inflammation leading to HIV-associated neurocognitive disorders. This report may aid in identification miRNAs that target viral RNAs and HIV/SIV specifically, as well as in identification of miRNAs that may be targets of new therapies to treat HIV.

## Background

Approximately 34 million people worldwide are currently infected with HIV according to the World Health Organization (http://www.who.int/features/qa/71/en/index.html). As a result of the development of Highly Active Antiretroviral Therapy (HAART), infected individuals are living longer, healthier lives than just two decades ago. Despite this success, patients living with HIV often suffer from complications associated with long-term infection such as cardiac and neurological disorders, in addition to side effects from antiretroviral drugs [1,2]. Along with HAART treatment, recently there has been research targeted at HIV eradication focused on therapeutic vaccine development [3], purging the latent reservoirs that harbor virus [4], and reducing the expression of the CCR5 co-receptors [5], cell-surface proteins, used by HIV for entry into cells. To this end, a better understanding of HIV-host interactions is needed.

The type I IFN response is a major host defense against virus infection [6,7]. HIV infection initiates a cascade of cytokine induction and innate immune signaling. In our SIV macaque model of HIV central nervous system (CNS) disease, analysis of plasma, cerebrospinal fluid, basal ganglia and parietal cortex shows distinct differences in innate immune responses between the periphery and the CNS. This is exhibited by the presence of viral RNA accompanied by induction of IFNβ and IFNβ-inducible MxA in the CNS as early as 4 days after infection [8]. CD4+ T cells are the main cells in the periphery that become infected, in contrast to the central nervous system (CNS), where CD14+ macrophages are the predominant productively infected cells [9-15]. Previous studies have demonstrated that circulating cells from the monocyte/macrophage lineage become infected and traffic to the brain where they induce cytokine signaling and also infect other macrophage lineage cells and astrocytes [6,16]. Many cytokines, including IFNβ, are produced by virus-infected or virus-exposed cells. Soluble IFNβ produced by infected cells binds to uninfected cells to signal through the type I IFN receptor, activating over 100 interferon-stimulated genes as well as antiviral proteins such as MxA [17]. Thus, the signal is amplified and a robust antiviral response is activated to prevent the spread of infection. Cellular restriction factors are another host defense against HIV infection. These include, but are not limited to, TRIM5α [18,19], APOBEC3G [20,21], SAMHD1 [22,23] and miRNAs [24,25].

miRNAs are small regulatory molecules that fine-tune levels of target mRNAs in the cell through binding to MREs in target mRNAs. Binding results in post-transcriptional repression caused by target mRNA degradation, translational inhibition and/or sequestration [26]. Each miRNA may have hundreds of predicted target mRNAs, resulting in regulation of expression for close to 60% of human mRNAs [27]. miRNAs regulate human physiology at the level of cell cycle and differentiation [28] and innate immune signaling and antiviral mechanisms [29-32], as well as yet to be identified genes and pathways. Their actions extend into roles in pathogenesis of several viruses including Hepatitis C [30,33], influenza [34,35], herpes viruses [36,37] and HIV.

miRNAs influence several stages of HIV-1 infection. Humans infected with HIV-1 have alterations in miRNA profiles [38] with differences in distinct miRNA populations in patients with varying levels of CD4+ T cell count/plasma HIV RNA copies [39] and elite suppressors [40-43], indicating involvement of miRNAs in response to virus infection. HIV modulates levels of several miRNAs at various time points after infection [44,45] and is also regulated by miRNAs in multiple ways [46]. Specific miRNAs target cytokines important in the immune response [47], as well as transcription factors, such as Cyclin T1 [48], which are necessary for transcription of viral genes. Some or all of miRNAs miRs-28, -125b, -150, -223 and -382 are reportedly involved in differential susceptibility of active and resting CD4+ T cells [49,50] and monocytes and macrophages [51,52] to HIV infection. Multiple reports suggest direct targeting of HIV-1 by host miRNAs [24,44,53-56]. Hariharan et al., used in silico analysis to identify target sites for host miRNAs in HIV-1 *vpr* (miR-149), *vif* (miR-324-5p), *vpu* (miR-378) and *nef*-LTR (miRs-29a and -29b) [53]. A follow-up study by the same group demonstrated miR-29a regulation of Nef expression and HIV-1 replication, and suggested that this was due to miRNA targeting the HIV-1 Nef transcript [24]. The miR-29 family was also reported to inhibit replication of HIV-1, demonstrating that the RNA-induced Silencing Complex (RISC) protein, Ago2, and P body protein, RCK/p54, directly interact with viral RNA in a miR-29a-dependent manner [54]. Nathans, et al. also showed that miR-29a binds the same MRE in HIV-1 as predicted by Hariharan et al. [53], and this site is conserved across HIV-1 subsets [54]. Sun et al. identified MREs for several miRNAs within HIV-1 and reported downregulation of miRs-21, 155, -29a, -29b and -29c, and an upregulation of miR-223 in response to HIV-1 infection in CD4+ T cells [55]. This group also reported only weak repression of a pNL4-3-Luc reporter by miRs-29a, -29b and -223, hypothesizing that this was due to a hairpin in this region of HIV-1 RNA sequence that interfered with RISC/miRNA binding [55]. In contrast to Sun et al., Schopman et al. found induction of miR-29a in several cell types in response to HIV-1 infection [44]. This difference may be due to time after infection when miRNA levels were measured or the specific cells that were used for the studies. Finally, Houzet et al. used anti-HIV-1 miR-326 as an example that sequence complementarity between a miRNA and its target mRNA correlates with inhibited expression of that target mRNA [56]. Together, these reports demonstrate modulation in levels of at least 13 human miRNAs during HIV-1 infection in various cell types. Two of these miRNAs (miRs-29a and -29b) have been validated by more than one study to have an effect on, or to directly target HIV-1 RNA transcripts [24,53-55].

We have developed a rapid and consistent SIV macaque model of HIV/AIDS and CNS disease in order to study the cellular and viral molecular events and pathogenesis during acute, asymptomatic and AIDS stage of disease [6-9,57]. We have shown both *in vivo* and *in vitro* that TNFα and IFNβ are induced during acute infection in SIV-infected macaques [7,8], and both cytokines regulate several miRNAs [30,32,58]. We demonstrate here that TNFα and IFNβ induce specific miRNAs at very early time points after SIV infection. SIV infection and cytokine stimulation of primary macrophages were used to dissect the mechanisms of miRNA induction, innate immune signaling and control of virus infection. We evaluated these miRNAs in regard to their effects on virus replication and mRNA levels, ability to target viral RNA sequences and modulation by innate immune signaling pathways. We provide evidence that the four miRNAs, miR-29a, -29b, -9 and -146a, are induced in macrophages during innate immune signaling and target the viral RNA, reducing virus replication and virus production.

## Results

### Predicted miRNA recognition elements (MREs) in SIV 3′ UTR

**Figure 1:**
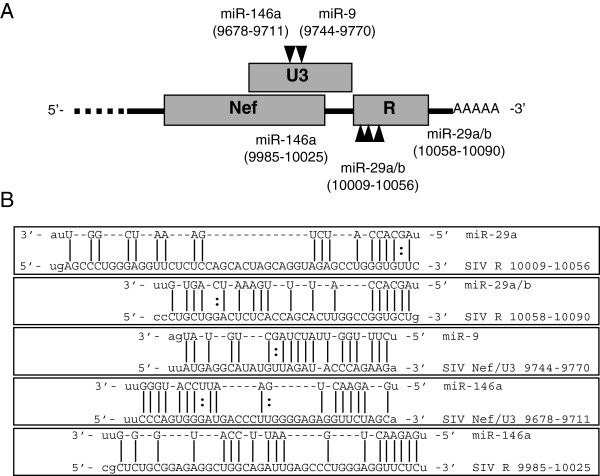
**Predicted miRNA binding sites within the 3′ UTR of SIV.** miRanda and RNAhybrid prediction programs identified MREs for miRs- 29a, -29b, -9 and -146a. **(A)** A graphic representation of the SIV 3′ UTR with predicted MREs. **(B)** Alignment of MREs within the SIV 3′ UTR generated from predictions.

miRNA target prediction programs [59,60] were used to identify potential miRNA binding sites within the 3′ untranslated region (UTR) of SIV 17E-Fr (Figure 1, Additional file 1: Table S1). Many miRNAs were identified that have predicted MREs in the SIV RNA 3′ UTR, and we focus here on miRs-29a, -29b, -9 and -146a, (Figure 1A and B). All four miRNAs contain promoter binding sites for transcription factors induced during innate immune signaling. miRs-29a and -29b are predicted to contain two ISRE (STAT1/STAT2 heterodimer induced by type I IFN signaling) GAS (STAT1 homodimer activated by IFNγ signaling) binding sites in the promoter [61] and are induced in response to IFNα/β and IFNγ. The miR-9 promoter contains an NF-κB binding site and is induced by TNFα in an NF-κB-dependent manner [58]. The miR-146a promoter is regulated by PU.1 and C/EBPα [62], transcription factors induced by innate immune signaling. In addition, the ability of miRs-29a and -29b to target HIV-1 transcripts has been supported by multiple studies [24,53-55]. The transcriptional activation of these miRNAs, in addition to the predicted binding sites in the SIV RNA sequence, suggests miRs-29a, -29b, -9 and -146a may be induced during the innate immune response and inhibit viral replication.

### Effects of miRs-29a, -29b, -9 and -146a on SIV production in primary macrophages

**Figure 2:**
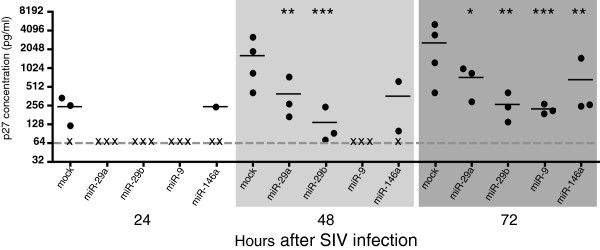
**Four miRNAs reduce levels of SIV virus production.** Primary macaque macrophages were transfected with 100 nM miRNA mimics, then infected with SIV (MOI 0.05) 24 hours after transfection. Supernatants were collected at 24, 48 and 72 hours post-infection and analyzed for virus production. Data shown is an average of at least 3 experiments for each miRNA at each time point. (x) indicates levels at or below the limit of detection, ~60 pg/ml (gray line). Results reported are p27 pg/ml values averaged from at least 3 separate experiments. All statistics reported are the result of a paired, two-sided t test performed on p27 values of time-point matched samples with untransfected controls. * = *p* < 0.05, ** = *p* < 0.01, *** = *p* < 0.001.

To determine if the miRNAs with predicted binding sites in the UTR of SIV have an effect on virus production, macaque macrophages were transfected or not with each of the miRNAs and infected with SIV twenty-four hours after transfection. Levels of virus released from cells were measured at 24, 48 and 72 hours post-infection (p.i.). At 24 hours p.i., miR-29a, -29b, -9 and -146a reduced virus production from ~250 pg/ml of p27 protein to below the limit of detection (~60 pg/ml) (Figure 2). There was a statistically significant decrease in virus production at 48 hours post-infection by miRs-29a and -29b. miR-9 continued to reduce p27 levels to below the limit of detection and miR-146a reduced p27 levels to below the limit of detection in cells from one experiment. Virus levels were decreased ~4-fold by miR-29a, ~15-fold by miR-29b and ~8-fold by miR-146a (Figure 2). A statistically significant decrease in virus production was maintained by all four miRNAs through 72 hours post-infection (Figure 2).

### miRNA-mediated reduction of full-length and spliced SIV RNA

**Figure 3:**
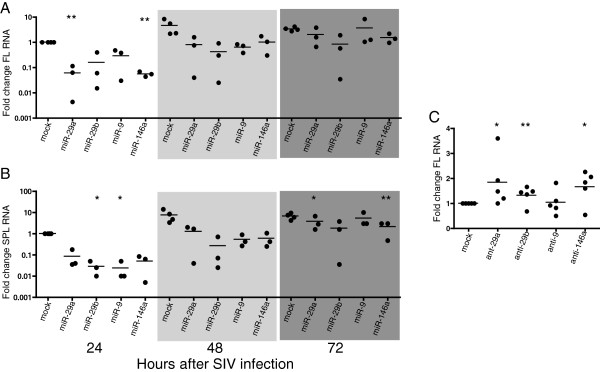
**Four miRNAs reduce levels of full length and multiply spliced viral RNAs.** Primary macaque macrophages were transfected with 100 nM of either miRNA mimics or antagonists, then infected with SIV (MOI 0.05) 24 hours after transfection. Cell lysates were collected at 24, 48 and 72 hours post-infection for miRNA experiments and 48 hours for miRNA antagonist experiments. Levels of full length **(A, C)** and spliced **(B)** RNA was measured by RT-qPCR. Values are expressed as fold change over SIV-only controls. Results are reported as percent of SIV-only control using the ΔΔCq method.

To investigate whether these miRNAs exert anti-SIV effects through transcript degradation or translation inhibition, total cellular RNA was isolated from the same cells used in Figure 2. Full-length and multiply-spliced (*tat/rev*) viral RNA levels were measured by RT-qPCR. All four miRNAs significantly decreased the levels of full-length (Figure 3A) as well as multiply-spliced (Figure 3B) SIV RNA. Full length and spliced RNA levels were most significantly reduced at 24 hours after infection when viral RNA abundance is lowest (Figure 3A and B, left panels). Experiments were also performed using 25 nM of miR-29b and the same reduction in SIV RNA levels was observed using this 4-fold lower miRNA concentration (data not shown).

We hypothesized that miRNA-specific antisense oligonucleotides (antagonists) that decrease the levels of available miRNAs would lead to an increase in viral RNA. Antagonists were transfected into macaque macrophages, and cell lysates were collected at 48 hours post-infection. We found that miRNA antagonists for miRs-29a, -29b and -146a significantly increased levels of SIV RNA when compared to SIV-infected untreated cells (Figure 3C). Inhibition of miR-9 did not increase viral RNA levels. This was presumably due to the fact that this miRNA is approximately 1000-fold less abundant than miRs-29a and -146a (data not shown), and inhibition by low copy number miRNAs may be inefficient [63,64].

### Effects of miRs-29a, -29b, -9 and -146a on expression of a luciferase reporter

**Figure 4:**
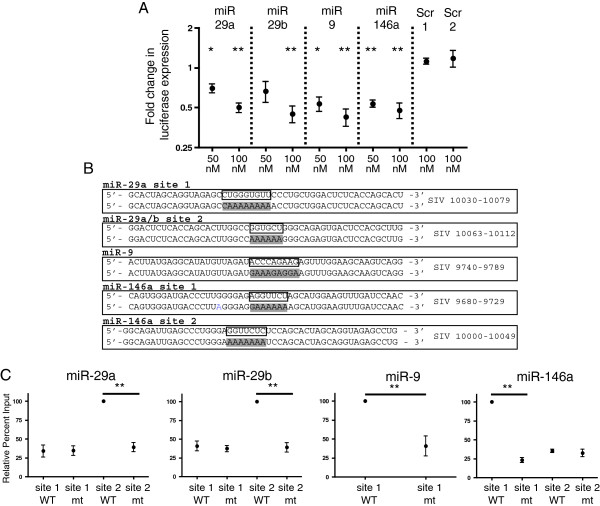
**miRs-29a, -29b, -9 and -146a reduce expression of a luciferase reporter and target predicted binding sites in the 3′ UTR of SIV. (A)** SIV *nef*/*U3*-*R* was cloned into psiCHECK2 plasmid (Promega). 293T cells were co-transfection with 50 nM or 100 nM of each miRNA or scrambled mimic and 100 ng of either psiCHECK+SIV or psiCHECK alone. Control samples were transfected with either psiCHECK or psiCHECK+SIV, and no miRNA mimics. Cells were harvested 24 hours after transfection. Data shown is an average of four experiments and represents miRNA effect on luciferase expression for psiCHECK+SIV over psiCHECK only. **(B)** WT and mutant biotin-labeled oligos corresponding to SIV RNA sequences containing predicted miRNA binding sites. WT oligo is show on top and mutant on bottom. Boxed bases on WT oligo denote miRNA seed binding site in SIV sequence. Mutant oligo highlighted bases denote the change in seed-binding region. Annotation is based on SIV sequence AY033146. **(C)** 100 pmol of individual WT or mt oligos were transfected into HeLa cells and lysates collected 24 hours after transfection. Oligos were pulled down using Streptavidin beads and assayed for binding to endogenous cellular miRNAs using Taqman RT-qPCR. Percent input was calculated for each sample (see methods) and data presented as (% input mt )/(% input WT) with % of input of WT set to 100%. In each experiment, the value of the oligo which pulled down the highest percentage of individual endogenous miRNA was set to 100% and binding by other oligos was compared to this value. Data shown is an average of 4 experiments. Statistics represent the comparison of percent of miRNA bound to WT oligo compared to percent of miRNA pulled down by corresponding mt oligo and are reported as a two-tailed t test assuming unequal variance.

After identifying predicted miRNA binding sites in the SIV 3' UTR and considering that all four miRNAs reduce levels of SIV RNAs and inhibit virus production, a luciferase assay was performed to examine the effects of the four miRNAs on expression of luciferase from a plasmid containing the predicted sites. Transfection of 293 T cells was done with a luciferase reporter plasmid alone, or a plasmid with genomic regions containing the SIV 3′ UTR. Luciferase expression was measured in the absence and presence of miRs-29a, -29b, -9 and -146a. Addition of each miRNA resulted in a dose-dependent inhibition of reporter gene expression (Figure 4A). Transfection with two different scrambled mimics had no effect on expression of the reporter gene (Figure 4A).

### Direct targeting of MREs in the SIV 3′ UTR

To determine whether the predicted SIV 3′ UTR MREs were bound by the predicted targeting miRNAs, we transfected into HeLa cells biotinylated 50-nucleotide RNA oligonucleotide molecules (oligos) that corresponded to wild-type (WT) or mutated (mt) MRE seed-binding SIV U3-R regions (Figure 4B). We calculated the difference in the percent of each miRNA precipitated by WT and mt oligos. Cells were lysed, and the biotinylated oligonucleotides were selected with strepavidin. Recovered miRNA was quantitated by RT-qPCR. The two predicted SIV 3′ UTR seed binding sites for miR-29 family members were included in two oligos corresponding to nucleotides (nts) 10030–10070 and 10063–10112 of the SIV RNA. The one predicted binding site for miR-9 was included in the oligo corresponding to nts 9740–9789 of the SIV RNA. The two predicted sites for miR-146a were included in two oligos corresponding to nts 9680–9729 and 10000–10049 of the SIV RNA (Figure 4B). miRs-29a and -29b bound to the site contained within nts 10063–10112 of the SIV genome, however, neither miR-29a nor miR-29b bound to the predicted site within nts 10039–10070 (Figure 4C). miR-9 bound to its predicted site contained within nts 9740–9789 (Figure 4C). miR-146a bound to the predicted site contained within nts 9680–9729, but did not bind to the predicted site contained within nts 10000–10049 (Figure 4C). These results demonstrate that there are functional binding sites for miRs-29a, -29b, -9 and -146a within the SIV 3′ UTR.

### Upregulation of mature anti-SIV miRNAs during SIV infection of primary macrophages

**Figure 5:**
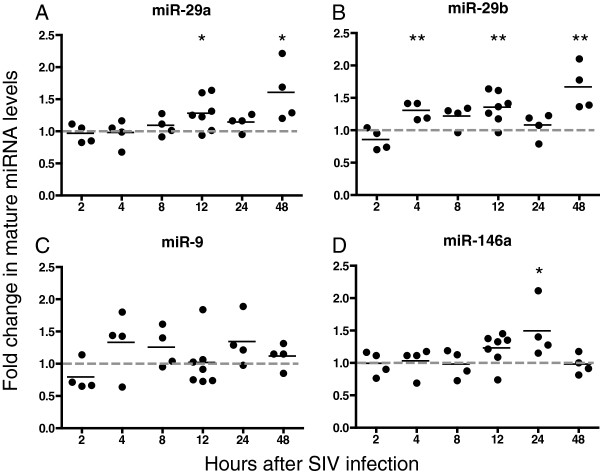
**SIV infection increases levels of mature miR- 29a (A), -29b (B), -9 (C) and -146a (D).** Macaque macrophages were infected with SIV (MOI 0.05). Cells were harvested at 2, 4, 8, 12, 24 and 48 hours after infection and RNA was isolated. Taqman miRNA RT-qPCR assays were used to measure levels of mature miRNAs. Results were normalized to U6, then to uninfected samples for the individual time points (ΔΔCq method). Values are expressed as fold induction of miRNAs over uninfected controls using the ΔΔCq method and data shown is an average of at least 3 experiments.

To examine whether SIV infection affects the levels of miRs-29a, -29b, -9 and -146a, each of these miRNAs was measured in primary macaque macrophages after SIV infection and compared with miRNA levels in uninfected time-point controls. Levels of all 4 miRNAs increased during infection (Figure 5). Primary macrophages were infected with SIV, and RNA was isolated at 2, 4, 8, 12, 24 and 48 hours post-infection. miR-29a and -29b levels increased together at 12 hours and by 50% and 60%, respectively, at the 48-hour time point (Figure 5A and B). Levels of miR-9 increased approximately 50-85% 4 hours after infection in three out of four animals (Figure 5C). There was also an increase in miR-146a levels at 12 and 24 hours after infection, with the largest average increase being 50% at 24 hours (Figure 5D). These data demonstrate an induction of mature miRNA levels during early stages of SIV infection and suggest that an increase in the levels of these miRNAs may contribute to the decrease in SIV replication and viral RNA levels that we have shown here.

### IFNβ-mediated upregulation of precursor and mature anti-SIV mRNAs

**Figure 6:**
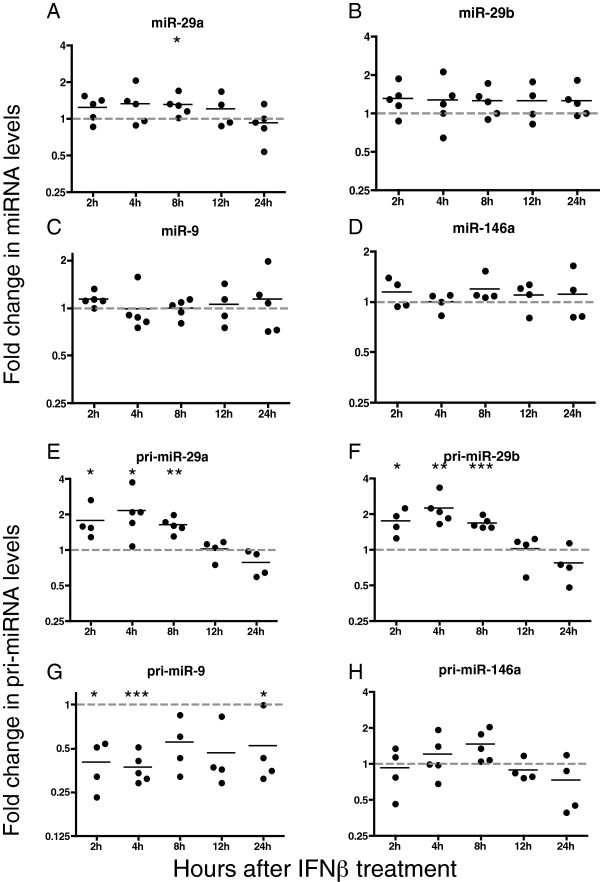
**IFNβ increases levels of mature miRNAs and primary miRNA transcripts in human macrophages.** Human macrophages were treated with 100 U/ml IFNβ and cells were harvested at 4, 8, 12 and 24 hours after treatment and RNA isolated. **(A-D)** Taqman miRNA RT-qPCR assays were used to measure levels of mature miRNAs. **(E-H)** Taqman pri-miRNA RT-qPCR assays were used to measure levels of primary miRNA transcripts. Results were normalized to U6. Values are expressed as fold induction of miRNAs over uninfected controls using the ΔΔCq method and data shown is an average of at least 3 experiments.

The increase in levels of miRs-29a, -29b, -9 and −146 (Figure 5) in response to SIV infection is unlikely to be caused directly by the virus, since the only viral proteins in the cells at this time are from the virion. IFNβ has been shown to modulate expression of miRNAs in our laboratory and by others [30,32], specifically miRs-29a and -29b [61]. We examined the effects of IFNβ treatment of cells on the expression levels of all four miRNAs. Human macrophages were used so that both the mature and pri-miRNAs could be measured (pri-miRNA assays have not been developed for macaque sequence). Macrophages were treated with IFNβ, and RNA was extracted at 4, 8, 12 and 24 hours after treatment. IFNβ caused a modest but consistent increase in the levels of miRs-29a and -29b at early time points, with no discernable difference observed for miRs-9 and -146a (Figure 6A-D). miR-29a was increased significantly at eight hours by 25% and miR-29b was increased similarly after stimulation with IFNβ (Figure 6A and B). Levels of miR-146a showed a similar trend at 8 hours (Figure 6D). This data shows that IFNβ induction early in infection leads to an increase in levels of miR-29a and -29b in primary human macrophages. IFNβ treatment produces similar results in primary macaque macrophages (Additional file 2: Figure S1A-D).

The increase in levels of mature miRNAs can be due to a decrease in miRNA turnover, an increase in transcription of precursor miRNAs (pri-miRNAs), or an increase in mature miRNA processing. To address at what level these miRNAs were being increased, pri-miRNA levels were measured in primary human macrophages at 2, 4, 8, 12 and 24 hours after IFNβ treatment (Figure 6E-H). Expression of pri-miRs-29a and -29b was significantly increased ~2-fold at 2, 4 and 8 hours after IFNβ treatment (Figure 6E and F). Coordinated regulation of these two miRNAs was expected, as they are part of the same transcript. Expression of miR-146a was increased 44% at 8 hours after treatment (Figure 6H). Transcription of miR-9 was significantly decreased at all time points (Figure 6G), explaining the lack of induction of the mature miRNA by IFNβ. An increase in the pri-miRNA indicates IFNβ stimulation caused an upregulation of the transcriptional activation of the three miRNA.

The canonical response to IFNβ was confirmed by measuring the levels of the interferon-stimulated gene, *mxa*. Both SIV infection and IFNβ stimulation significantly increased *mxa* expression (Additional file 3: Figure S2A,B), and IFNβ also reduced viral RNA levels (Additional file 3: Figure S2C). These results demonstrate canonical signaling of pathways downstream of IFNβ in our primary macrophage system.

### TNFα signaling drives expression of IFNβ and anti-SIV miRNAs

**Figure 7:**
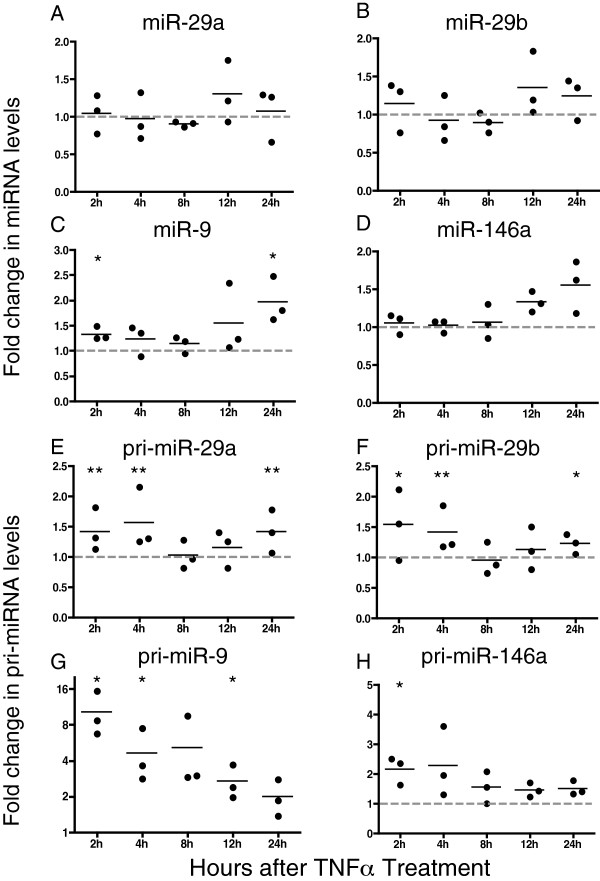
**TNFα increases levels of mature miRNAs and primary miRNA transcripts in human macrophages.** Human macrophages were treated with 20 ng/ml TNFα. Cells were harvested at 2, 4, 8, 12 and 24 hours after treatment and RNA isolated. **(A-D)** Taqman miRNA RT-qPCR assays were used to measure levels of mature miRNAs. **(E-H)** Taqman pri-miRNA RT-qPCR assays were used to measure levels of primary miRNA transcripts. Results were normalized to U6. Values are expressed as fold induction of miRNAs over uninfected controls using the ΔΔCq method and data shown is an average of at least 3 experiments.

In primary macrophages, IFNβ stimulation modulates levels of pri- and mature miRs-29a and 29b, and pri-miR-146a (Figure 6A-H). Like IFNβ, TNFα is induced during the acute phase of SIV infection as part of the innate immune response [8], and TNFα has been shown to increase expression of IFNβ and interferon-stimulated genes [65]. TNFα stimulation of primary macrophages resulted in an increase in the IFNβ-stimulated gene, *mxa* (Additional file 4: Figure S3), demonstrating regulation of this pathway by TNFα. To test the effect of TNFα stimulation on miRNA levels, primary macrophages were treated or not with TNFα and cells harvested at 2, 4, 8, 12 and 24 hours after treatment. TNFα stimulation resulted in an increase in miR-29a and -29b levels by an average of 30% and 35%, respectively, at 12 hours (Figure 7A and B). miR-9 levels increased significantly at 2 and 24 hours after treatment by an average of 40% and 90%, respectively (Figure 7C). Levels of miR-146a increased maximally by an average of 55% at 24 hours (Figure 7D). Macaque macrophages responded similarly to stimulation with TNFα (Additional file 5: Figure S4A-D).

TNFα stimulation also induced transcriptional expression of all four miRNAs (Figure 7E-H). pri-miR-29a expression increased by ~45% 2 hours after treatment, with a maximum increase of 55% 4 hours after treatment (Figure 7E). Similarly, pri-miR-29b expression increased maximally 2 hours after treatment by 55% and remained elevated by ~40% 4 hours (Figure 7F). There was a dramatic 10-fold increase of pri-miR-9 2 hours after treatment with TNFα. This increase was maintained at 5-fold 4 hours after treatment, and 2-fold through the 12 hour time point (Figure 7G). pri-miR-146a expression increased by 2-fold 2 and 4 hours after treatment. An approximate 50% increase was maintained through the 12-hour time point (Figure 7H).

## Discussion

The identification of miRNAs as regulators of gene expression has dramatically changed the understanding of post-transcriptional regulation of cellular and viral genes. A number of viruses encode miRNAs in their genome that regulated cellular genes required for replication [66]. We have previously shown that miRNAs in plasma of SIV-infected macaques provide a signature of infection and progression to CNS disease, demonstrating that SIV infection affects miRNA expression *in vivo*[67]. In this study, we demonstrate for the first time that four miRNAs directly bind to the U3 region of the SIV RNA. Further, all four miRNAs, miR-29a, -29b, -9 and -146a, controlled SIV virus production and replication by decreasing SIV full length and spliced *tat/rev* RNAs in infected primary macrophages. In addition we provide evidence that these four miRNAs are transcriptionally regulated by the innate immune response, specifically, TNFα and type I IFN. We have shown previously that virus replication in macrophages increases both of these cytokines and thus, it appears that these miRNAs are part of the innate antiviral immune response to SIV.

The protein products of spliced *rev* and *tat* transcripts are critical early in infection for progression to later stage and productive infection. The 3′ UTR of SIV contains MREs for all four miRNAs, miRs-29a, -29b, -9 and -146a. All SIV RNAs share the same 3′ UTR sequence and therefore, these four miRNAs have the capability to target all full-length and spliced viral transcripts for degradation. In order to have a significant impact on virus infection and the propagation of virus, one would hypothesize that miRNAs would target the early multiply-spliced genes, preventing the progression of virus replication to the productive stage. In addition, we hypothesized that the levels of the miRNAs would be high in the cell or would increase within hours following infection in a cell or be upregulated in response to early cytokines in bystander cells, preventing virus spread to other cells. Levels of the anti-SIV miRNAs studied here increased as early as 4 hours post-infection, and miRs-29a, -29b and -146a were significantly induced by 24 and 48 hours after infection, respectively. From these results, we see that viral infection in macrophages upregulates miRNAs that regulate the virus.

It has been shown for virus infections, including HIV and SIV, that infection of cells, particularly macrophages, leads to the induction of innate immune responses, type I IFN and TNFα [8,17]. To determine whether the increased levels of the four miRNAs that reduce virus infection were induced by the cellular response to infection, we used INFβ and TNFα to examine the induction of mature as well as pri-miRNAs. INFβ and/or TNFα, two cytokines that are made in response to SIV infection in macrophages increased the four miRNAs. INFβ increased miRs-29a and -29b as early as 2 hours after treatment. TNFα, in contrast increased levels of miRs-29a, -29b and -146a at 12 hours suggesting that its effect was due to stimulation of type I IFN. TNFα may have a direct effect on miR-9 since the miRNA was significantly increased at 2 hours and appears not to be modulated through the type I IFN response. Overall, levels of the mature form of these miRNAs increased approximately 50% over control.

While these increases were modest, we found them consistently, and it is important to remember that viral infection drastically alters the pre-existing miRNA-target network in many ways. Upon infection, host miRNAs that may have multiple host targets are provided with many copies of a new, viral target. The abundance of cellular transcript targets may also be modulated. Since the number of targets of a specific miRNA and the affinity of the targeting miRNA for each target together influence the magnitude of regulation along with the abundance of the miRNA itself, large fold changes or indeed any modulation of targeting miRNA are not necessarily prerequisites for regulation of an evolutionarily novel target. Further investigations of the entire network are needed to establish the necessary stoichiometries for effective regulation. We would also note that this miRNA/target network is even more complicated *in vivo*, as levels of the characterized miRNAs may be altered in cell types other than macrophages. These miRNAs may traffic between cells [68] and may be recycled within a given cell [69], thereby enhancing their effects.

A second important finding is that innate immune responses induced these miRNAs at the transcriptional level. During this early time after infection, SIV spliced RNA encoding Rev and Tat are produced, and this is likely the time when miRNA levels are most efficiently targeting viral transcripts to reduce virus replication. Expression of each miRNA transcript is controlled by specific transcription factors. The promoters of miRs-29a, -29, -9 and -146a all contain one or more binding sites for NF-κB [58,70,71], a transcription factor induced by innate immune signaling. These reports show that miRs-9 and -146a are induced by TNFα and induction of miR-146a is dependent on the three NF-κB binding sites found in its promoter. In cancer cells, TLR signaling and NF-κB activation were shown to suppress expression of miRs-29a and -29b [70], but the promoter for these two miRNAs contains binding sites for several other transcription factors related to innate immune signaling.

While miRs-29a, -29b, -9 and -146a did not evolve to target SIV, they reduced virus production via direct interaction with specific sequences in the 3′ UTR of SIV RNA. In addition, these and other miRNAs may modulate virus infection directly as well as indirectly. For example, miR-29a has been shown to target IFNγ [72]. A report by Chiang et al., demonstrates direct binding of Cyclin T1 by miR-29b and [73], a transcription factor necessary for replication of HIV-1. miR-9 is induced by TLR signaling and NF-κB activation and regulates expression of the NF-κB p50 subunit [58,74]. miRNA-146a is a negative regulator of innate immunity and overexpression results in downregulation of type I IFN in PBMCs due to targets within IRAK1 and TRAF6 [71,75-78]. miR-146a was also reported to target and inhibit expression of CXCR4 [79], a cell surface receptor used by HIV-1 and certain strains of SIV. miRNAs are differentially expressed at various times in different tissues. Furthermore, individual miRNAs may have different factors controlling maturation as well as rates of degradation. More investigation is needed to elucidate the connections between IFNβ/TNFα signaling and our observed modulation of transcription of these miRNAs. What is becoming clear is that this miRNA response reflects another downstream antiviral innate immune effector.

Several lines of evidence suggest that miRNAs may be useful as therapeutic inhibitors of HIV-1 infection. These data include: 1) a relationship between differences in miRNA profiles and cell type susceptibility to HIV-1 infection [49-52]; 2) dysregulation of miRNAs during HIV-1 infection [39] and 3) direct miRNA targeting of HIV-1 RNA sequences [54-56]. These reports show the potential for miRNAs to be given therapeutically as potent inhibitors of HIV-1 infection. The main obstacle is delivery of these molecules to a desired tissue or cell population. Several groups have reported nanoparticle delivery of antiretrovirals and siRNAs in HIV-infected cells *in vitro* as well as in mouse models of neuroAIDS [80]. These methods appear promising for delivery to specific cell and tissue types, as well as only to cells that are infected. Obad et al. demonstrated using short LNA antagomiRs to target entire miRNA families [81], and this strategy could be used to inhibit miRNAs that are overexpressed during infection and increase either virus infection or contribute to pathogenesis. We show here that miRs-29a, -29b, -9 and -146a are four anti-SIV miRNAs that have the potential to be used as therapeutic agents against SIV infection.

## Conclusion

To our knowledge, this study is the first report linking viral as well as innate immune modulation of miRNAs with direct targeting of viral RNAs and inhibition of virus production. The significant role of miRNAs in the pathogenesis of HIV is underscored by the abundance of reports demonstrating miRNA effects on disease progression as well as effects of virus on miRNA expression. The early induction of miRs-29a, -29b, -9 and -146a caused by SIV and the significant inhibition of virus production very early in infection suggests potential for using these as well as other miRNAs for treatment of HIV in addition to other infectious diseases. Our macaque model of SIV provides an ideal model for testing delivery and efficacy of treatment with miRNAs. Future strategies include using anti-SIV miRNAs or miRNAs that target transcription factors and other proteins necessary for replication of HIV and SIV. Targeted delivery to infected cells or individual cell types may reduce negative off-target effects suffered by many patients currently on HAART.

## Methods

### Ethics statement

Animal studies were approved by the Johns Hopkins University Institutional Animal Care and Use Committee (Protocol # PR09M296) and conducted in accordance with the Weatherall Report, the Guide for the Care and Use of Laboratory Animals, and the USDA Animal Welfare Act.

### miRNA prediction programs

The SIV 3′ UTR sequence, consisting of *nef/U3-R* (SIV/17E-Fr nucleotides 9462–10155), was analyzed for miRNA binding sites. Results from miRanda [59] and RNAhybrid [60] were compiled to identify predicted miRNA binding sites within the SIV 3′ UTR. The minimum free energy cutoff for miRNA consideration was set at −20 kcal/mol, and the free energy of each miRNA/target site interaction is in Additional file 1: Table S1.

### Luciferase assays

The SIV 3′ UTR (*nef/U3-R*, SIV 17E-Fr nts 9458–10160) was cloned into the psiCHECK-2 vector (Promega) 3′ of a Renilla luciferase reporter gene. 293 T cells were plated at 80,000 per well of a 24-well plate. Cells were approximately 90% confluent 24 hours later for co-transfection of individual miRNA mimics and either psiCHECK-2 plasmid plus SIV insert or psiCHECK-2 plasmid with no insert. Controls were transfected with either psiCHECK-2 or psi-CHECK-SIV 3′ UTR, but no miRNA mimics. Cells were harvested 24 hours later and assayed for luciferase expression using the Renilla Luciferase Assay System (Promega) and measured on (Fluoroskan Ascent FL, Thermo Scientific). The raw luciferase value of each sample was normalized to total protein for that sample to control for well-to-well differences in cell number. Firefly luciferase was not used for normalization as all four miRNAs reduced levels of this control reporter. Several individual plasmids were co-transfected to use as transfection controls but were not able to be included because expression of all was modulated by some or all of miRs-29a, -29b, -9 and -146a. Experiments were repeated replacing the miRNA mimics with scrambled mimics (scrambled #1 Ambion mirVana miRNA mimic Negative Control #1, scrambled #2 Sigma Mission miRNA Negative Control 2).

### Macrophages

#### Isolation and culture

Macaque macrophages were obtained from rhesus and pigtail macaque donors. Human macrophages were obtained from leukopacks from anonymous donors of the Johns Hopkins Hospital HATS donation center. Whole blood was diluted with HBSS, loaded onto a Ficoll gradient and centrifuged for 30 minutes at 2000 rpm. Plasma was removed and PBMCs were removed from the interface of plasma and Ficoll. Cells were washed in HBSS and pelleted (1500 rpm for 10 minutes) twice prior to red blood cell lysis (15 ml of 150 mM NH_4_CL, 10 mM KHCO_3_ and 1 mM EDTA for 15 minutes at 37°C). 35 ml of HBSS was added, and PBMCs were centrifuged to remove red blood cell lysis buffer. Cells from individual donors were plated separately (donor samples were not pooled) at 4×10^6^ cells per well in 12-well plates or 10×10^6^ cells per well in 6-well plates and cultured for 7 days in medium containing M-CSF and 20% autologous serum. One half of the total volume of medium was replaced on day 3. On day 7, cells were washed 3 times with PBS to remove non-adherent cells. The medium was replaced the same as above, only with 10% serum. Infection and treatments were all performed on day 8 after plating.

#### miRNA transfection and SIV infection

Rhesus or pigtail macaque macrophages were approximately 90% confluent on day eight post-plating when transfected with 100 nM of individual miRNAs or miRNA antagonists (Qiagen) for 6 hours using Lipofectamine 2000 (Invitrogen/Life Technologies) diluted in OptiMEM Reduced Serum Medium (Life Technologies) as per manufacturer’s instructions. Mock transfections included Lipofectamine 2000 and OptiMEM, but no miRNA mimic.. Twenty-four hours later, cells were infected with macrophage tropic SIV 17E-Fr [GenBank:AY033146.1] at an MOI of 0.05 for six hours. Following infection, cells were washed 3 times with PBS, and fresh medium was added. Supernatants and cells were collected at 24, 48 and 72 hours post infection to measure virus production and viral RNA levels, respectively. All results shown are the average of at least three separate experiments.

#### IFNβ and TNFα treatment

Day 8 pigtail macaque macrophages were approximately 90% confluent when treated with either 100 U/ml IFNβ (PBL Interferon Source) or 20 ng/ml TNFα (R&D Systems, human 210-TA-010, macaque 1070-RM-025). Total RNA was isolated from control and treated samples at 2, 4, 8, 12 and 24 hours post-infection. All results shown are the average of at least three separate experiments.

### miRNA isolation

All RNA isolation was performed using the mirVana miRNA Isolation Kit (Ambion). Cells were harvested in 600 μl Lysis/Binding Buffer and eluted with 100 μl water. Samples were treated with 2 μl TURBO DNase (Ambion) for 45 minutes at 37°C. Samples were re-extracted using the mirVana miRNA Isolation Kit cleanup protocol and eluted with 100 μl water.

### Analysis of SIV p27

200 μl of supernatant was used for p27 assays (Zeptometrix). The assay was performed following an overnight incubation of samples in lysis buffer at 37°C. p27 levels were determined based on the manufacturer’s provided standard. All results shown are the average of at least three separate experiments.

### RT-qPCR

Results are reported as fold change using the ΔΔCq method. All statistics reported represent 2-tailed t tests assuming unequal variance, performed on ΔΔCq values of time-point matched samples with untreated/uninfected controls. All results shown are the average of at least three separate experiments.

#### SIV transcripts

250 ng of RNA was used for reverse transcription cDNA synthesis (Superscript III, Invitrogen/Life Technologies). RT-qPCR was performed as described previously using primers and probes specific for SIV *gag* and *tat/rev* transcripts [82]. Quantification cycle (Cq) values were normalized to an average of the GAPDH and18S Cq values as well as to time-point controls.

#### Taqman RT-qPCR – mature miRNAs

10 ng of total RNA was used for reverse transcription cDNA synthesis using the Taqman microRNA Reverse Transcription Kit (Applied Biosystems) and Taqman assays for individual miRNAs. 5 μl of cDNA was used for RT-qPCR with individual miRNA Taqman assays. Cq values were normalized to a U6 internal control as well as to time-point controls.

#### Taqman RT-qPCR – pri-miRNAs

100–250 ng of total RNA was used for reverse transcription cDNA synthesis using the High Capacity cDNA Reverse Transcription Kit (Applied Biosystems). 4 μl of cDNA was used for RT-qPCR with Taqman precursor miRNA assays for individual pri-miRNAs. Cq values were normalized to a U6 internal control as well as to time-point controls.

### RNA immunoprecipitation

HeLa cells were plated using 200,000 cells per well of a 6-well plate and 24 hours later were approximately 90% confluent when transfected with Lipofectamine 2000 (Invitrogen) with 400 pmol of a biotinylated 50-nucleotide WT or mutant RNA oligo corresponding to the predicted regions of binding for individual miRNAs (see Figure 5A). Twenty-four hours after transfection cells were fixed in 0.5% formaldehyde for 15 minutes at room temperature. Cross-linking was stopped by 5 minute incubation at room temperature in 0.25 M glycine, pH 7. Fixed cells were lysed in modified RIPA buffer [82] for 30 minutes at 4°C. Lysed cells were centrifuged for 10 minutes at 14,000 rpm at 4°C. 5% of total sample volume was taken for input. Lysates were incubated for 30 minutes at room temperature with 100 μl of streptavidin beads (Dynabeads MyOne Streptavidin C1, Life Technologies) to pull down endogenous miRNAs bound to the biotinylated oligos. Beads were washed 3 times with 500 μl RIPA Buffer. After reverse cross-linking (5 minutes at 65°C in 100 μl of 95% formamide, 10 mM EDTA pH 8.0), 500 μl of mirVana Binding/Lysis Buffer was added to the supernatant and RNA was extracted using the mirVana miRNA Isolation Kit (Ambion). 10 μl of total RNA was used in Taqman miRNA-specific qPCR primer/probe assays to detect specific miRNAs bound to individual oligos containing predicted miRNA binding sites. Percent of pull down by each oligo was determined and normalized by the Percent Input Method (http://www.invitrogen.com/site/us/en/home/Products-and-Services/Applications/epigenetics-noncoding-rna-research/Chromatin-Remodeling/Chromatin-Immunoprecipitation-ChIP/chip-analysis.html).

### Statistical analyses

For mRNA and miRNA comparisons that only consisted of two groups (uninfected/untreated versus infected/treated) a paired, two-sided t test was performed on the well-specific observations (relative to the control average) at each time point. Each miRNA was statistically evaluated as the duplicate- or triplicate-averaged difference from C(q) U6 or 18S and GAPDH relative to control. Statistical significance, p-value <0.05, indicates an effect of intervention. * = *p* < 0.05, ** = *p* < 0.01, *** = *p* < 0.001.

## Competing interests

The authors declare that they have no competing interests.

## Authors’ contributions

JMS, KWW and JEC conceived of and managed the study. JEC provided resources. JMS performed the experiments. JMS and KWW analyzed the data and wrote the manuscript. PMT analyzed data and guided statistical analyses for all experiments. KWW and JEC contributed to revision of the manuscript. All authors read drafts and approved the final manuscript.

## Supplementary Material

Additional file 1: Table S1 Binding energies for each miRNA site within the SIV LTR sequence, predicted by miRanda and RNAhybrid. The minimum binding energy threshold was set to −20 kcal/mol. Click here for file

Additional file 2: Figure S1 **A-D**. IFNβ increases levels of mature miRNAs in macaque macrophages. Macaque macrophages were treated with 100 U/ml IFNβ. Cells were harvested at 4, 8, 12 and 24 hours after treatment and RNA was isolated. Taqman miRNA RT-qPCR assays were used to measure levels of mature miRNAs. Results were normalized to U6. Values are expressed as fold induction of miRNAs over untreated controls using the ΔΔCq method and data shown is an average of at least 3 experiments. Click here for file

Additional file 3: Figure S2 SIV infection and IFNβ stimulation increases *mxa* levels. IFNβ decreases SIV RNA levels. Macaque macrophages were infected with SIV **(A)**, infected with SIV and treated with IFNβ **(C)** and primary human macrophages were treated with IFNβ **(B)**. RT-qPCR using sequence-specific primers and probe for *mxa* was used to measure *mxa* and SIV RNA levels. Results were normalized to 18S. Values are expressed as fold induction of *mxa* over uninfected/untreated controls using the ΔΔCq method and data shown is an average of at least 3 experiments. Click here for file

Additional file 4: Figure S3 TNFα induced expression of *mxa* in primary macaque and human macrophages. Macaque **(A)** and human **(B)** macrophages were treated with 20 ng/ml macaque or human TNFα. RNA was isolated at 2, 4, 8, 12 and 24 hours after treatment. Sequence-specific primers and probe for *mxa* were used for RT-qPCR. Results were normalized to 18S. Values are expressed as fold induction of *mxa* over untreated controls using the ΔΔCq method and data shown is an average of at least 3 experiments. Click here for file

Additional file 5: Figure S4 **A-D**. TNFα increased levels of mature miRNAs in macaque macrophages. Macaque macrophages were treated with 20 ng/ml TNFα. Cells were harvested at 4, 8, 12 and 24 hours after treatment and RNA isolated. Taqman miRNA RT-qPCR assays were used to measure levels of mature miRNAs. Results were normalized to U6. Values are expressed as fold induction of miRNAs over untreated controls using the ΔΔCq method and data shown is an average of at least 3 experiments. Click here for file
