# Supplementary material for: SIV replication is directly downregulated by four antiviral miRNAs
Source: Retrovirology. 2013 Aug 29;10:95. doi: 10.1186/1742-4690-10-95 (PMC3766675; doi:10.1186/1742-4690-10-95)
Supplement: Additional file 1: Table S1 — Binding energies for each miRNA site within the SIV LTR sequence, predicted by miRanda and RNAhybrid. The minimum binding energy threshold was set to −20 kcal/mol. [file 1742-4690-10-95-S1.pdf]

Table S1

	Kcal/Mol	
Site	miRanda	RNAHybrid
miR-29a 10009-10056	-22.07	-
miR-29a 10058-10090	-24.08	-20.5
miR-29b 10058-10090	-21.32	-20.3
miR-9 9744-9770	-22.6	-21.9
miR-146a 9678-9711	-29.47	-26.7
miR-146a 9985-10025	-23.89	-
