# Supplementary figures and images for: SIV replication is directly downregulated by four antiviral miRNAs
Source: Retrovirology. 2013 Aug 29;10:95. doi: 10.1186/1742-4690-10-95 (PMC3766675; doi:10.1186/1742-4690-10-95)

Figure S1

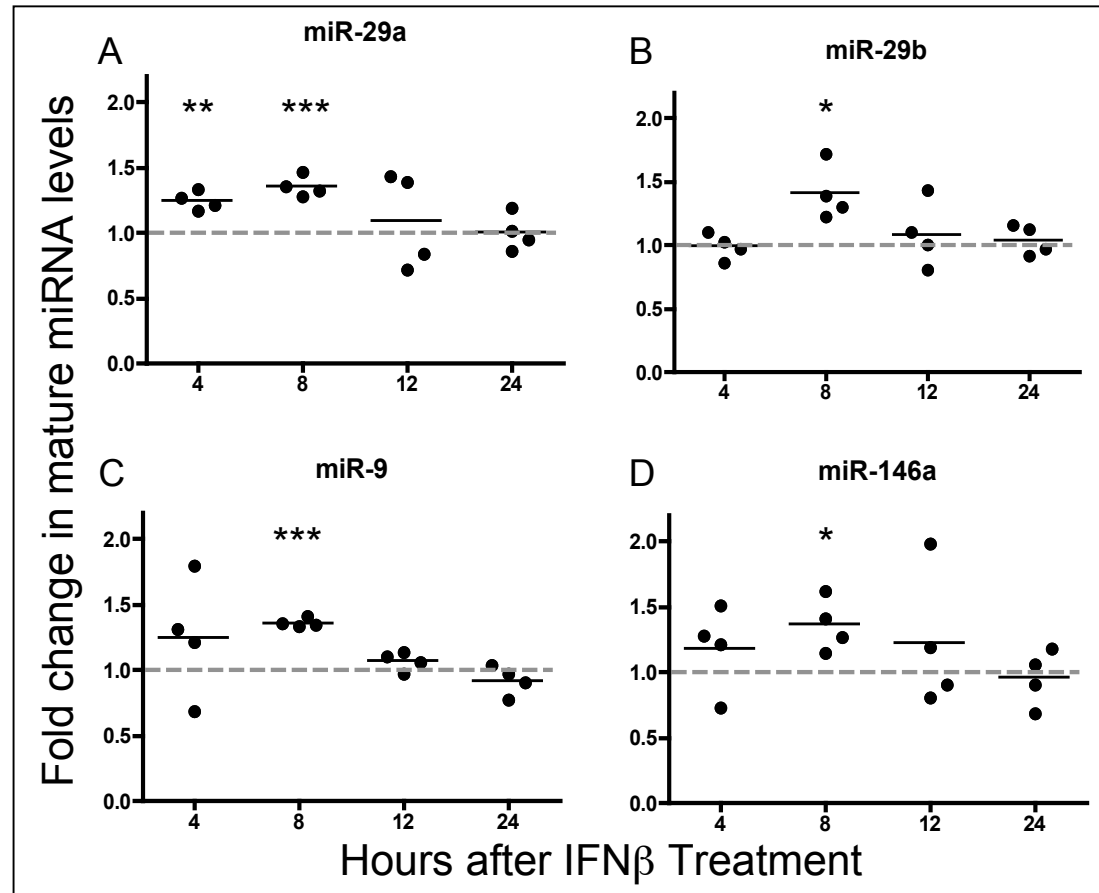

Supplement: Additional file 2: Figure S1 — A-D. IFNβ increases levels of mature miRNAs in macaque macrophages. Macaque macrophages were treated with 100 U/ml IFNβ. Cells were harvested at 4, 8, 12 and 24 hours after treatment and RNA was isolated. Taqman miRNA RT-qPCR assays were used to measure levels of mature miRNAs. Results were normalized to U6. Values are expressed as fold induction of miRNAs over untreated controls using the ΔΔCq method and data shown is an average of at least 3 experiments. [file 1742-4690-10-95-S2.pdf]

Figure S2

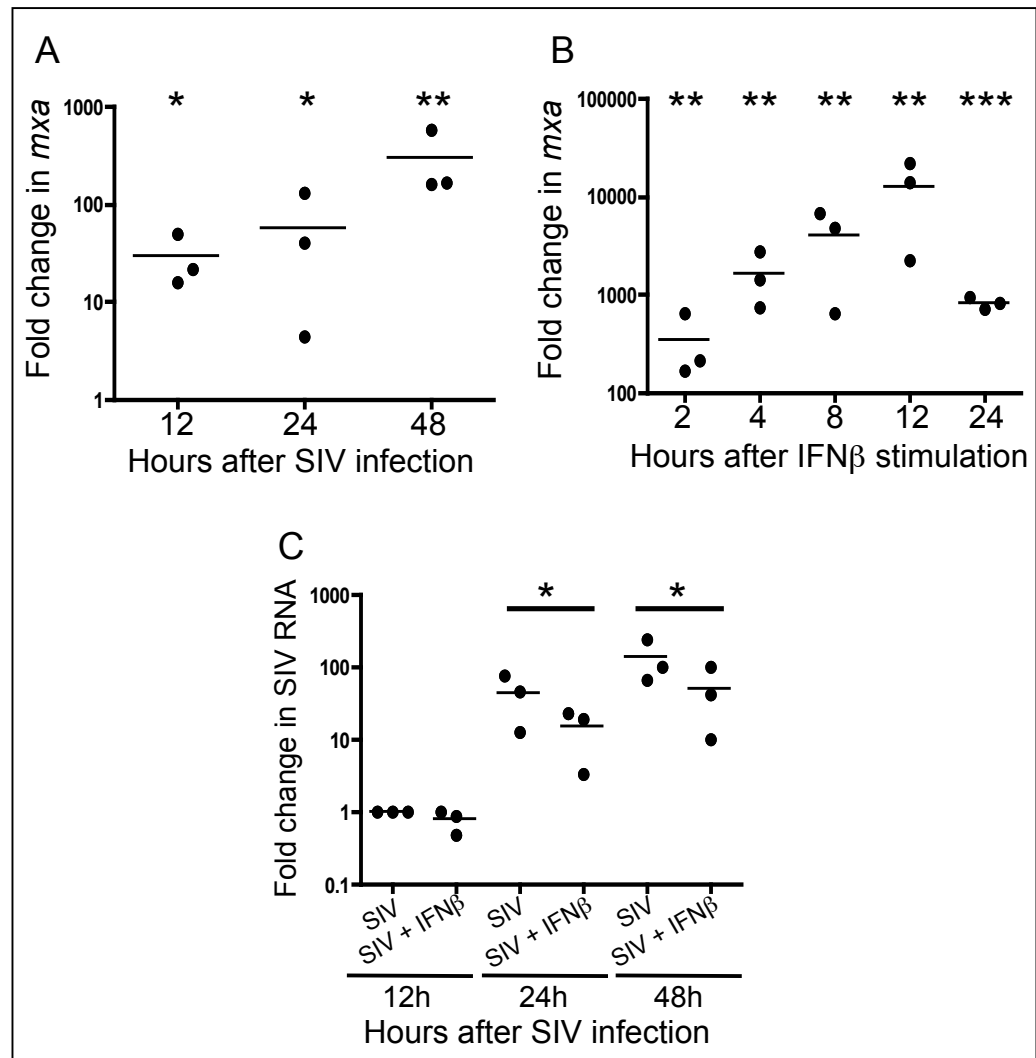

Supplement: Additional file 3: Figure S2 — SIV infection and IFNβ stimulation increases mxa levels. IFNβ decreases SIV RNA levels. Macaque macrophages were infected with SIV (A), infected with SIV and treated with IFNβ (C) and primary human macrophages were treated with IFNβ (B). RT-qPCR using sequence-specific primers and probe for mxa was used to measure mxa and SIV RNA levels. Results were normalized to 18S. Values are expressed as fold induction of mxa over uninfected/untreated controls using the ΔΔCq method and data shown is an average of at least 3 experiments. [file 1742-4690-10-95-S3.pdf]

Figure S3

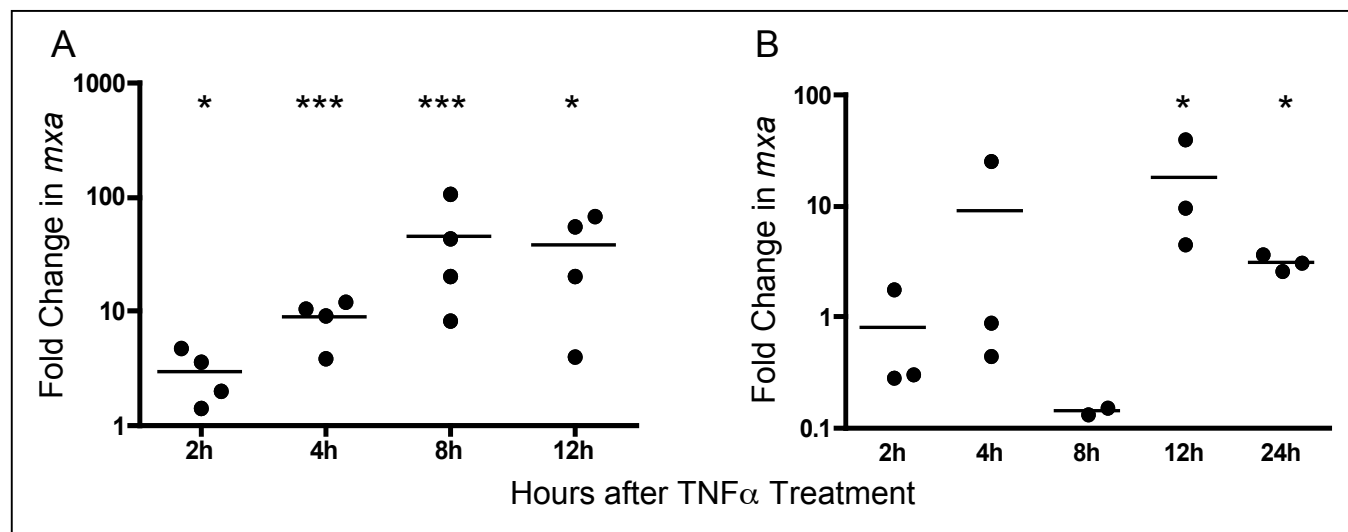

Supplement: Additional file 4: Figure S3 — TNFα induced expression of mxa in primary macaque and human macrophages. Macaque (A) and human (B) macrophages were treated with 20 ng/ml macaque or human TNFα. RNA was isolated at 2, 4, 8, 12 and 24 hours after treatment. Sequence-specific primers and probe for mxa were used for RT-qPCR. Results were normalized to 18S. Values are expressed as fold induction of mxa over untreated controls using the ΔΔCq method and data shown is an average of at least 3 experiments. [file 1742-4690-10-95-S4.pdf]

Figure S4

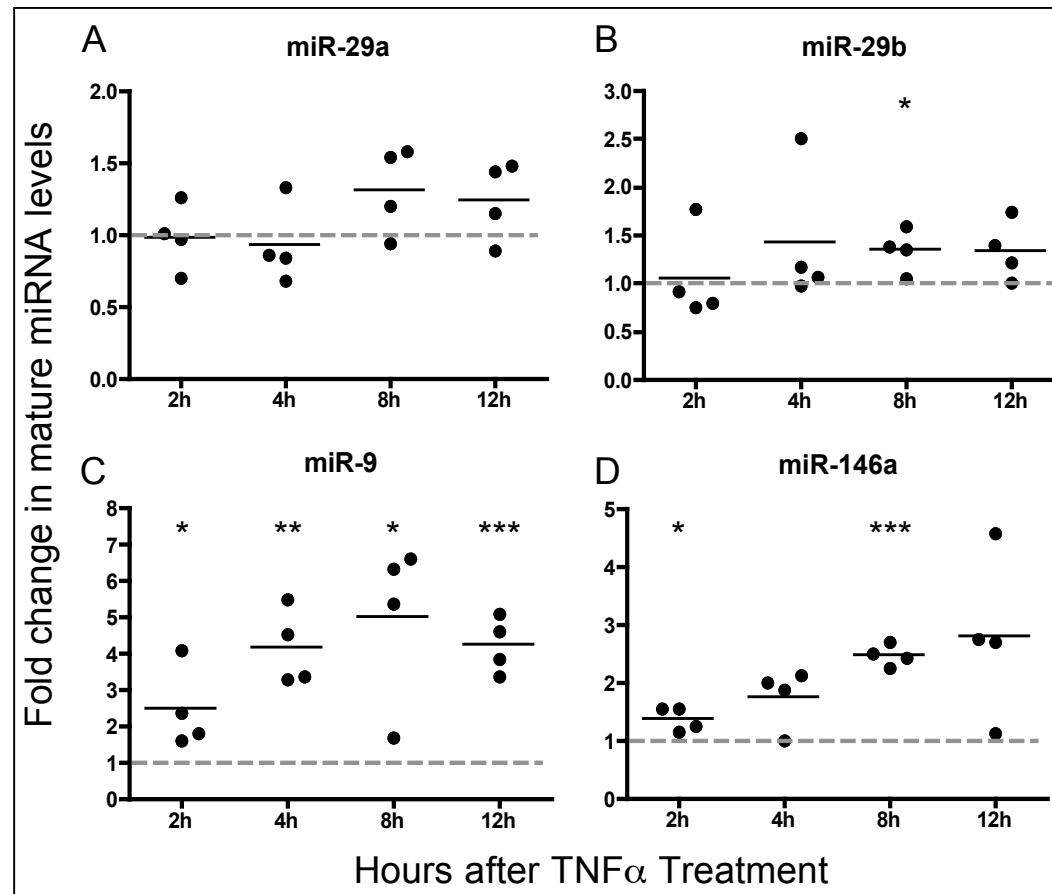

Supplement: Additional file 5: Figure S4 — A-D. TNFα increased levels of mature miRNAs in macaque macrophages. Macaque macrophages were treated with 20 ng/ml TNFα. Cells were harvested at 4, 8, 12 and 24 hours after treatment and RNA isolated. Taqman miRNA RT-qPCR assays were used to measure levels of mature miRNAs. Results were normalized to U6. Values are expressed as fold induction of miRNAs over untreated controls using the ΔΔCq method and data shown is an average of at least 3 experiments. [file 1742-4690-10-95-S5.pdf]
